# γ-Radiation-assisted molecular template route: a new hybrid path for facile synthesis of size-dependent optical properties of ZnS quantum dots

**DOI:** 10.1098/rsos.250692

**Published:** 2025-06-11

**Authors:** Sanju Francis, Nisha Kushwah, Vishwanadh Bathula, Kedarnath Gotluru

**Affiliations:** ^1^ Radiation Technology Development, Bhabha Atomic Research Centre, Mumbai, Maharashtra, India; ^2^ Department of Chemistry, Bhabha Atomic Research Centre, Mumbai, Maharashtra, India; ^3^ Materials Science Division, Bhabha Atomic Research Centre, Mumbai, Maharashtra, India

**Keywords:** radiation, ZnS QDs, photoluminescence

## Abstract

ZnS is a benign and multi-utility semiconductor with absorption in the UV–vis region of the energy spectrum. Nevertheless, the synthesis of ZnS quantum dots (QDs) with tunable optical properties and a reasonable photoluminescent quantum yield (PLQY) adopting a new hybrid method is highly recognized. The present study involves the simple synthesis of self-capped wurtzite ZnS QDs employing a hybrid method comprising a single-source molecular precursor (SSMP), 2-(dimethylamino)ethanethiolate of zinc(II), and γ-radiation followed by elucidation of the formation mechanism of self-capped ZnS QDs. Here, the SSMP has been γ-irradiated in a solution to yield ZnS QDs of varying size at different radiation doses. The crystal structure, elemental composition, shape and optical properties of pristine self-capped ZnS QDs were assessed by powder X-ray diffraction, energy dispersive X-ray spectroscopy, electron microscopy, UV–vis, photoluminescence and diffused reflectance spectroscopy, respectively. The size-tailored emission maximum and optical band gaps were tweaked to a tune of 417–537 nm and 4.17–4.23 eV by altering the γ-radiation dose with PLQYs realized in the range of 10–24%. Lifetimes of these samples are in the range of 1.69–2.68 and 6.82–34.88 ns for the fast- and slow-decaying components, respectively.

## 1. Introduction

ZnS, a wide band gap group 12−14 binary semiconductor, finds extensive applications in solar cells [1], optoelectronics [2] and UV photodetectors [3]. The former occurs either in thermodynamically stable cubic zinc blende or in high-temperature stable form hexagonal wurtzite crystal systems [4]. Nevertheless, the wurtzite form of ZnS reverts back to zinc blende ZnS upon cooling. Hence, stabilization of the wurtzite ZnS under ambient conditions has been realized either through doping of metal ions [5] or producing ZnS in nanoform by heat treatment under different conditions [6] or the ZnO template method [7].

The applicability of ZnS can be further broadened for bioimaging [8], biosensing [9] and display optoelectronics [10] through quantization of ZnS. The quantization of the respective bulk counterparts of any semiconducting material significantly upgrades its bulk properties owing to the confinement of electrons and holes in a region as small as the excitation Bohr radius of the bulk material. The confinement of charge carriers leads to their recombination, leading to amelioration of optical properties. Quantum dots (QDs) exhibit fascinating fluorescence properties. For instance, the former unveils size and phase-dependent emission properties facilitating them to excite in a wide range of wavelengths below the fluorescence wavelength. Further, photoluminescent quantum yield (PLQY) and emission properties are dependent on particle dimension, preparation method and doping element. These properties further widen their prospects in electroluminescence and solar cells [11].

Until now, quite a few methods have been adopted for the synthesis of ZnS QDs [12–14]. Among them, solution-based techniques [15–21] have an edge over other methods owing to a greater number of variable reaction parameters, which can be tuned to obtain different sizes, shapes, phases and hence the wide range of interesting luminescence properties. Of these, the single-source molecular precursor (SSMP) approach has always been versatile owing to its interesting merits [22]. The molecular proximity of M and S (where M = Metal and S = Sulfur) through pre-existing M–S bonds in SSMPs can promote single-phasic metal sulfide, thereby evading the formation of other impurities or else that are bound to exist or form if metal salts are used as precursors. Therefore, tri-n-octyl phosphine oxide (TOPO)-capped ZnS nanoparticles have been isolated by using ethyl(diethyldithiocarbamato)zinc(II) as a precursor [23]. Similarly, Liu [24] and Sun *et al.* [25] reported the synthesis of 2.8−6.6 and 5 nm ZnS nanoparticles by thermolysis and microwave synthesis, respectively, by employing the same precursor under different conditions. Although there are a few reports that discuss self-capped nanomaterials [26–28], synthesis protocol for size-dependent tunable luminescent ZnS QDs without any external passivating agent/surfactant is rare to the best of our knowledge. Additionally, the optical properties of semiconductor materials are predisposed by their crystalline nature, imperfections and chemical purity. Thus, it is intriguing to synthesize ZnS QDs and investigate their optical properties isolated by a new hybrid method in combination with SSMP and the γ-radiation-assisted method. Furthermore, radiolytic synthesis of nanoparticles is a green method because additional use of high-boiling solvents acting as reducing agents is not required, while the SSMP method can account for scalable synthesis of QDs. In short, the new hybrid method presented in this study can deliver a green and facile route for scalable synthesis at room temperature. Moreover, the γ-radiation-assisted method is a room temperature process; hence, undesired reaction side products formed by high temperature can be avoided in comparison to other heating methods such as thermolysis and pyrolysis.

Therefore, encouraged by previous investigations on dimethylaminoethane chalcogenolate systems from our group [6] and a quest for a new hybrid method, the production of self-capped wurtzite ZnS QDs as a model system has been demonstrated employing 2-(dimethylamino)ethanethiolate of zinc(II), 2-(dimethylamino)ethanethiolate of zinc(II), as SSMP. Note that ZnS QDs prepared in this account were surface passivated by *in situ* generated organic fragments of the ligand employed for the synthesis of SSMP. Furthermore, the formation mechanism of self-capped ZnS QDs has also been elucidated for the first time. Moreover, optical properties associated with ZnS QDs generated by this hybrid method have also been evaluated. Additionally, size-dependent photoluminescent emission (PLEm) and optical band gap tunability ranging from 417 to 537 nm and 4.17−4.23 eV of ZnS QDs with PLQYs in the range of 10–24% have been realized. It is noteworthy to mention that 24% is the highest quantum yield (QY) for ZnS QDs devoid of any inorganic shell growth obtained through γ-radiation-assisted molecular precursor-mediated synthesis. In addition, this type of hybrid method can be extended to the preparation of other materials where innate defects developed in the nanomaterials owing to γ-radiation can facilitate the improved performance, especially in defect-driven applications.

## 2. Experimental

### 2.1. Material and methods

HSCH_2_CH_2_NMe_2_.HCl and Zn(OAc)_2_.2H_2_O were purchased from commercial sources (Sigma-Aldrich). Solvents (methanol, acetonitrile and dichloromethane) were purified prior to their use in the reactions and measurements.

### 2.2. Characterization techniques

Elemental analyses were carried out on a Thermo Fischer Flash EA1112 CHNS elemental analyser. The ^1^H NMR spectra were recorded on a Bruker Avance-II spectrometer operating at 300 MHz, respectively. Chemical shifts are relative to internal chloroform peak for ^1^H NMR spectra. Powder X-ray diffraction (p-XRD) patterns were obtained on a Philips PW-1820 powder diffractometer using CuK_α_ radiation. Average crystallite size (*D*) was estimated from the Scherrer formula *D* = 0.9 *λ*/*β* cosθ, where *λ* is the X-ray wavelength and *β* is the full width at half maximum (FWHM). Energy dispersive X-ray spectroscopy (EDS) measurements were performed on the Oxford INCA E350 instrument. Transmission electron microscope (TEM), Tecnai G2 T20, operational at accelerating voltages up to 200 kV, was used for the TEM studies. The TEM samples were prepared by dropping the sample dispersed in a suitable solvent on a carbon-coated copper grid. UV–vis spectra were recorded in dichloromethane on a UV–vis Jasco V-630 spectrophotometer.

Edinburgh Instruments FLSP 920 system, with a 450 W Xe lamp and 60 W microsecond flash lamp, was employed for carrying out luminescence measurements at room temperature. A BaSO_4_-coated integrating sphere was used for QY measurements. All emission and excitation spectra were corrected for the detector response and the lamp profile, respectively. The resolution of emission measurements is 5 nm.

Time-resolved fluorescence measurements were performed by employing a time-correlated single-photon counting set-up (IBH, UK). In the current study, 339 and 455 nm LED (approx. 800 ps, 1 MHz repetition rate) was engaged as the excitation source and a TBX-4 detector for fluorescence detection. A re-convolution method was employed to examine the observed decays using a proper instrument response function obtained by replacing the sample with a suspension of TiO_2_ in water. Time resolution of the instrument is better than 50 ps in the current set-up. The fluorescence decays were expressed as a sum of exponentials, as mentioned in equation (2.1), where *I*(*t*) is fluorescence intensity varying with the time and *A_i_* and *τ_i_* are the amplitude and the fluorescence lifetime of the excited state for the *i*^th^ component of the fluorescence decay:


(2.1) $$I(t)=\Sigma A_{i}\cdot exp(-t/\tau_{i}).$$

The goodness of the fits and the following bi- and tri-exponential decays are justified by the reduced *χ*^2^ values and the weighted residuals distribution among the data channels. For a suitable fit, the *χ*^2^ value is near 1, and the weighted residuals are arbitrarily spread over the data channels.

### 2.3. Synthesis of precursor

**Scheme 1.**
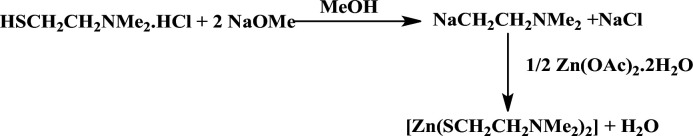
Synthesis protocol for the preparation of 2-(dimethylamino)ethanethiolate of zinc(II).

The precursor, 2-(dimethylamino)ethanethiolate of zinc(II) (scheme 1), was synthesized as reported in the literature [6] (electronic supplementary material).

### 2.4. γ-Radiation-assisted synthesis of ZnS quantum dots

Purified water from the Super-Genie water system was used in all the experiments. The conductivity was less than 0.055 μS cm^−1^. All glassware was washed with chromic acid followed by careful rinsing with deionized water. The pure ZnS precursor (13.7 mg) was dissolved in a CH_3_CN/water (25 ml each) mixture. The solution was purged by N_2_ to remove the dissolved oxygen, prior to irradiation. The solution was divided into three glass vials and irradiated in a gamma chamber (GC 5000, BRIT, India) using ^60^Co source to graded doses of 50 (A1), 150 (A2) and 200 kGy (A3) at a dose rate of 6.6 kGy h^−1^. The resultant solutions were washed with excess methanol and centrifuged to get pure ZnS QD samples.

## 3. Results and discussion

### 3.1. Synthesis and characterization of self-capped ZnS quantum dots

2-(dimethylamino)ethanethiolate of zinc(II) (scheme 1), was synthesized by adding a solution of Zn(OAc)_2_.2H_2_O in methanol to a freshly prepared methanolic NaSCH_2_CH_2_NMe_2_ (prepared by the addition of a methanolic solution of HSCH_2_CH_2_NMe_2_.HCl to a freshly prepared NaOMe in methanol in 1 : 2 molar ratio) under inert atmosphere and purified by recrystallization in dichloromethane (scheme 1). The pure complex was characterized by elemental analysis and by NMR (^1^H and ^13^C{^1^H}) spectroscopy.

The choice of 2-(dimethylamino)ethanethiolate of zinc(II) (scheme 1), for the γ-radiation-assisted molecular precursor-mediated synthesis of ZnS QDs without the use of any surface passivating agent is based on our previous experience with 2-(dimethylamino)ethaneselenolate complex of cadmium(II) [29], wherein it was found that 2-(dimethylamino)ethaneselenolate ligand passivates the surface of CdSe QDs in *in situ* fashion without any additional surface passivating or capping agents like oleylamine or TOPO. Furthermore, the relatively lower decomposition temperature of 2-(dimethylamino)ethanethiolate derivatives and decent affinity of the amine group for zinc also enable the surface passivation of QDs. With this perception of self-capping of QDs by 2-(dimethylamino)ethanethiololate derivatives, ZnS QDs have been synthesized by γ-radiolytic decomposition of 2-(dimethylamino)ethanethiolate of zinc(II) (scheme 1) at different γ-radiation doses at a constant rate of dose. The ZnS QDs with varying sizes were separated at different doses of γ-radiation impinged on the reaction mixture to assess the tunability of optical properties.

**Figure 1.**
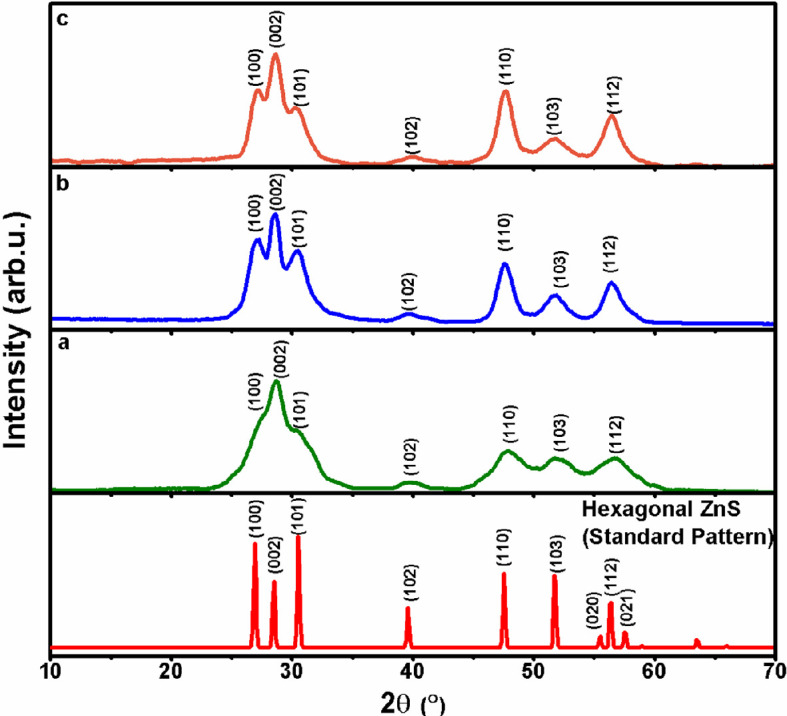
XRD patterns of ZnS QDs isolated at a graded subjected dose of a) 50 (sample A1), b) 150 (sample A2) and c) 200 kGy (sample A3) on a solution of 2-(dimethylamino)ethanethiolate of zinc(II) (**1**) in CH_3_CN/H_2_O at a dose rate of 6.6 kGy h^−1^ overlaid on standard hexagonal ZnS XRD pattern (ICSD file no. 67453/JCPDS file no.79-2204).

Phase purity and crystal microstructure of ZnS QDs (A1, A2 and A3) were examined by the p*-*XRD technique (figure 1). The p*-*XRD peak profiles of A1, A2 and A3 exhibit Bragg’s peaks at 2θ = 26.92, 28.53, 30.51, 39.60, 47.55, 51.78 and 56.39°, which can be indexed to the reflections from (100), (002), (101), (102), (110) (103) and (112) endorsing the wurtzite phase of ZnS (Inorganic Crystal Structure Database (ICSD) no. 67453; figure 1). The appearance of wide peaks in p-XRD points out that particle dimensions are in the nano regime. The average crystallite sizes of QDs as determined by the Scherrer equation are 2.1, 3.0 and 6.8 nm, respectively. The p-XRD results were further validated by the elemental composition of these samples by EDS analysis (table 1), which authenticates a nearly 1 : 1 atom% ratio of Zn : S (electronic supplementary material, figures S1–S3) corresponding to ZnS stoichiometry.

**Table 1.** Average particle size calculated from p-XRD, Brus expression and TEM, and EDS analyses of A1, A2 and A3 samples of ZnS QDs.

ZnS QD samples	average crystallite size from p-XRD (nm)	particle size by Brus expression (nm)	particle size by TEM (nm)	EDS analyses of Zn : S (atom%)	*E*_*g*_ from Tauc’s plot (in eV)
A1	2.1	3.1	3.9	50.91 : 49.09 (1.04 : 1.00)	3.95
A2	3.0	4.0	5.7	50.52 : 49.48 (1.02 : 1.00)	3.89
A3	6.8	5.6	9.5	50.29 : 49.71 (1.01 : 1.00)	3.84

**Figure 2.**
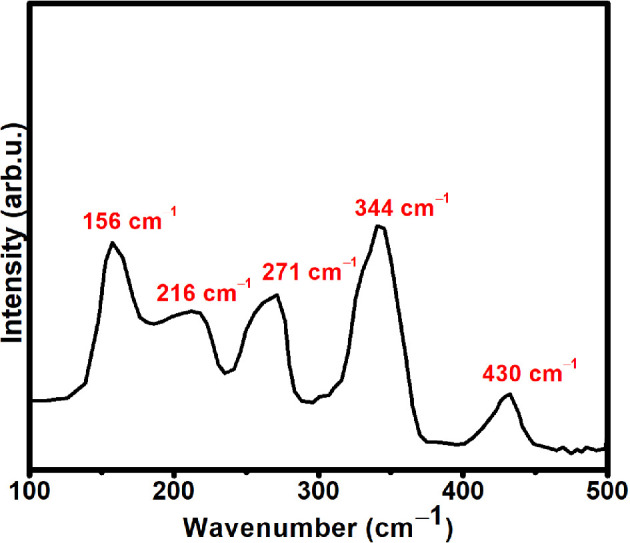
Raman spectrum of a representative ZnS QD.

The Raman spectrum of one of the representative ZnS QD samples was obtained in the 100−500 cm^−1^ range and is shown in figure 2. Wurtzite ZnS fits in the C_6V_ point group and gives rise to nine optic branches with irreducible representation as *Γ*_opt_ = A_1_ + E_1_ + 2E_2_ + 2B_1_. The B_1_ modes are both infrared (IR) and Raman inactive. On the contrary, polar A_1_ and E_1_ modes are both Raman and IR active, while non-polar E_2_ modes are only Raman active [30]. Raman spectrum of the sample unveils broad peaks at 156, 216, 271, 342 and 431 cm^−1^. The first two peaks can be attributed to two-phonon processes [30]. A peak at 216 cm^−1^ can be marked for longitudinal acoustic overtones along M-K line of Brillouin zone [31], while the peak at 271 cm^−1^ corresponds to A_1_ transverse optical (TO) mode. The peak at 343 cm^−1^ can be assigned as A_1_ longitudinal optical mode, while the peak at 430 cm^−1^ can be allotted to second-order (TO-transverse acoustic)X scattering [32].

**Figure 3.**
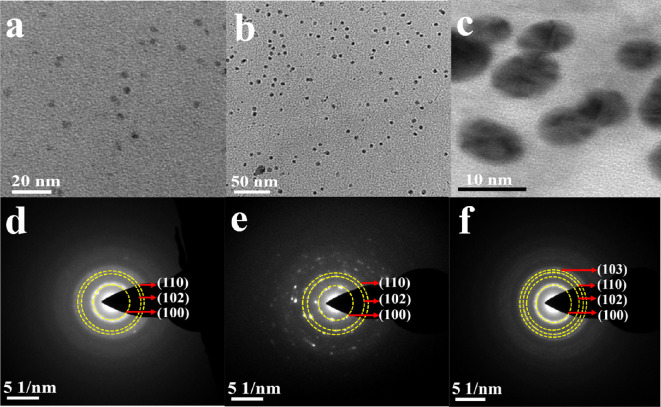
(a-c) TEM images, and (d-f) SAED patterns of ZnS QDs isolated at a graded subjected dose of 50 (sample A1), 150 (sample A2) and 200 kGy (sample A3) on a CH_3_CN/H_2_O solution of 2-(dimethylamino)ethanethiolate of zinc(II) (**1**) at a dose rate of 6.6 kGy h^−1^.

The morphological and microstructural/phase determination studies of all ZnS QD samples (A1, A2 and A3) were investigated by TEM and selected area electron diffraction (SAED) (figure 3). TEM images showcased spherical ZnS QDs of sizes 3.9, 5.7 and 9.5 nm, respectively, for A1, A2 and A3 minutes. This endorses the appearance of an exciton peak in the UV–vis spectrum of these samples indicating quantum confinement of these particles. The average crystallite size calculated by p-XRD differs from the size determined by TEM. This may be reasoned owing to the fact that particle size estimated by p-XRD gives the size of the smallest crystallite size, while TEM gives the size of particle that might contain many small crystallites.

SAEDs of A1, A2 and A3 show concentric circles consistent with (100), (102), (110) and (103) planes of the wurtzite phase of ZnS (ICSD no. 67453). The circular features unveiled in SAED of A1, A2 and A3 indicate a polycrystalline nature.

The spherical ZnS QDs formation of varying sizes with surge in the irradiation time for a fixed dose rate may be explained as follows. The creation of any QDs or nanocrystals is an interplay of nucleation and growth processes. Since the shape of the particles is spherical, the growth of nuclei is isotropic, which might have arisen as a result of the involvement of both surface diffusion and precursor desorption.

### 3.2. Mechanism for the formation of self-capped ZnS quantum dots

The possible mechanism for the formation of self-capped ZnS QDs by the decomposition of 2-(dimethylamino)ethanethiolate of zinc(II) can be elucidated on similar lines that have been explained for the decomposition of thiols [6]. Here, the decomposition of 2-(dimethylamino)ethanethiolate of zinc(II) is anticipated to yield ZnS through a reduction reaction of 2-(dimethylamino)ethanethiolate of zinc(II) with e^−^ (aq) to yield ^•^S(CH_2_CH_2_NMe_2_)_2_ and ZnS followed by the reduction of ^•^S(CH_2_CH_2_NMe_2_)_2_ to ^•^S(CH_2_CH_2_NMe_2_)_2_ and subsequent β-hydrogen elimination of SCH_2_CH_2_NMe_2_ (equations (3.1)–(3.3)). Dimethyl ethylene diamine, H_2_C=CHNMe_2_, has been acting as a capping agent through the surface passivation of ZnS QDs.


(3.1) $$\left(Zn(SCH_{2}CH_{2}NMe_{2}{)}_{2})+\bar{e}(aq\right)⟶\;{\;}^{\cdot }S(CH_{2}CH_{2}NMe_{2}{)}_{2}+ZnS,$$

(3.2) $${\;}^{\cdot }S(CH_{2}CH_{2}NMe_{2}{)}_{2}+\bar{e}(aq)⟶S(CH_{2}CH_{2}NMe_{2}{)}_{2},$$

(3.3) $$S(CH_{2}CH_{2}NMe_{2}{)}_{2}⟶2H_{2}C=CHNMe_{2}+H_{2}S$$

**Figure 4.**
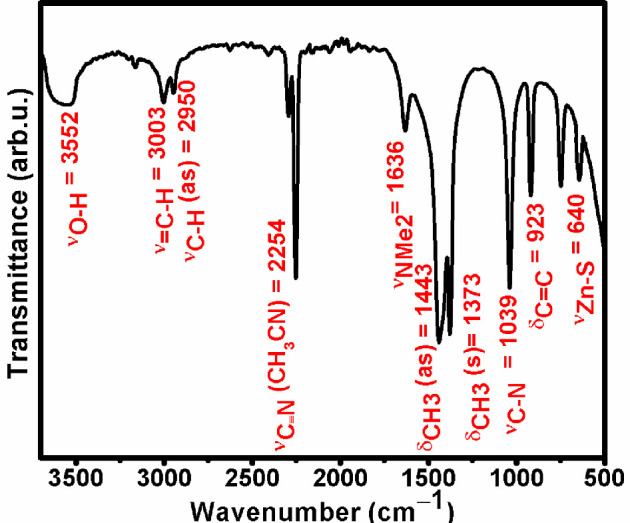
IR spectrum of a representative ZnS QD sample.

The formation of H_2_C=CHNMe_2_ is further confirmed by examining the surface of ZnS nanostructures by Fourier transform infrared (FT-IR) spectroscopy (figure 4). FT-IR spectra of ZnS nanostructures unveiled distinctive IR modes corresponding to ν_=CH_ (3003 cm^−1^), ν_C–H_ (asymmetric stretching frequency 2950 cm^−1^), ν_C–N_(NMe_2_) (1636 cm^−1^), ν_C–N_(=C N) (1039 cm^−1^), δ_C–H_(CH_3_) (asymmetric bending mode (1443 cm^−1^) and symmetric bending mode (1373 cm^−1^)), δ_C=C_ (923 cm^−1^) and ν_Zn–S_ (640 cm^−1^), demonstrating the presence of H_2_C=CNMe_2_, which might be capping ZnS QDs through the lone pairs of nitrogen atoms of the NMe_2_ group. Furthermore, the occurrence of ν_O–H_ (H_2_O) (3554 cm^−1^) and ν_C°N_ (CH_3_C°N) (3554 cm^−1^) stretches confirms the presence of water and acetonitrile molecules.

### 3.3. Optical properties and quantum yield of self-capped ZnS quantum dots

**Figure 5.**
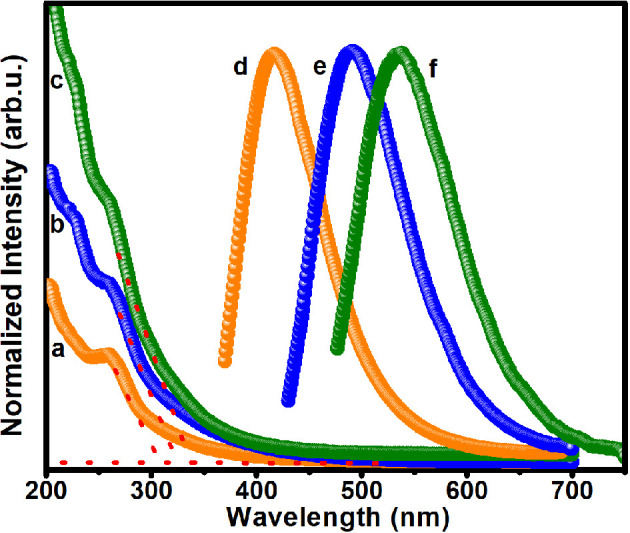
UV–vis absorption (a,b,c) and PLEm (d,e,f) spectra of self-capped ZnS QDs isolated at a graded subjected dose of 50 (sample A1), 150 (sample A2) and 200 kGy (sample A3) on a solution of 2-(dimethylamino)ethanethiolate of zinc(II) (**1**) in acetonitrile at a dose rate of 6.6 kGy h^−1^.

Bulk ZnS, a niche visible light absorber, has an optical band gap of 3.68 and 3.79 eV [33] depending on whether the crystal structure is zinc blende or wurtzite. Therefore, optical properties of ZnS QD samples (A1, A2 and A3) including UV−vis absorption, photoluminescence (PL) (photoluminescent excitation and PLEm) and optical band gap properties were investigated. The absorption spectra (figure 5) of the colloidal solutions of samples A1, A2 and A3 in chloroform unveil excitonic features, consistent with exciton transition. The absorption maximum is considerably blue-shifted compared to bulk ZnS (approx. 344 nm) owing to the quantum confinement.

Photoluminescent studies of ZnS QDs (A1, A2 and A3) have been performed by exciting the samples at their respective absorption maximum. The PL emission profiles of these samples exhibit tunable emission maximum ranging from 417 to 537 nm (figure 5). The PL emission spectra of A1, A2 and A3 display broad emission peaks with maximum at 417, 491 and 537 nm, respectively, with an excitation maximum of 355, 420 and 469 nm (electronic supplementary material, figure S4). A steady shift in emission maximum might be a combined effect of increasing particle size and trapped states as a result of incomplete surface passivation. The emission maximum observed at 417 nm may be attributed to the transition of electrons from shallow defect states near the conduction band to the sulphur vacancies existing close to the valence band [34]. The broad spectral width (FWHM of approx. 96–111 nm) of PL designates slightly broad size distribution of QDs. The Stokes shifts, as estimated from the difference between UV–vis exciton maximum and emission maximum, emerge in the range of 1.82−2.37 eV. The large Stokes shifts observed for the broad emission peaks indicate the participation of trap states in the emission process [35].

PLQYs of A1, A2 and A3 ZnS QD samples are 24, 14 and 10%, respectively (table 2). In general, QY values decrease with the increase in particle size owing to the fact that the probability of electron-hole interactions diminishes.

**Table 2.** Optical properties of A1, A2 and A3 samples of self-capped ZnS QDs.

sample identity	UV–vis exciton max. (nm)	UV–vis. *λ*_onset_ (nm)	PL Ex. max. (nm)	PL max. (nm)	FWHM of PL peaks (nm)	Stokes shift (eV)	*E*_*g*_ from *λ*_onset_ (in eV)	*E*_*g*_ from Tauc’s plot (in eV)	QY (%)
A1	259	306	355	417	96	1.82	4.05	3.95	24
A2	261	321	420	491	101	2.22	3.87	3.89	14
A3	265	331.6	469	537	111	2.37	3.74	3.84	10

The PLQYs may be enhanced further either by passivating the QDs surface with different types of ligands or by guarding the QD surface with an inert inorganic shell of low phonon energy.

### 3.4. Optical band gap measurements of self-capped ZnS quantum dots

The optical band gaps of self-capped ZnS QDs were estimated by using Tauc’s plot using equation (3.4) [36]:


(3.4) $$(\alpha hv{)}^{1/2}=\;A(h-E_{g}).$$

The band gap values of ZnS samples (A1, A2 and A3) could be derived by extrapolating the tangent to the *x*-axis (hν) from the plot of (αhν)^1/2^ versus energy.

**Figure 6.**
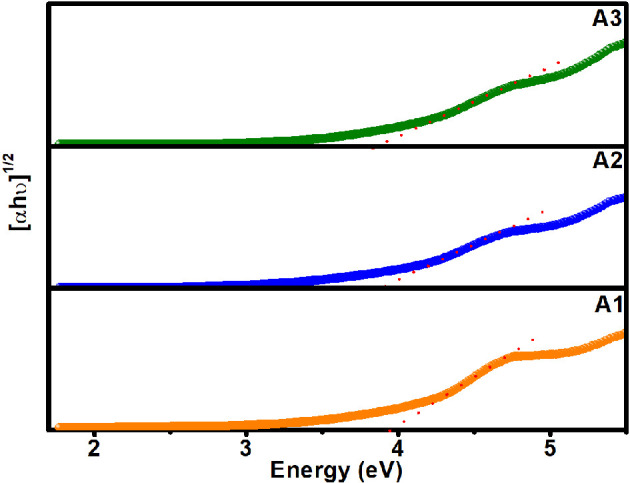
Tauc’s plots of (αhν)^1/2^ versus energy for determining optical direct band gaps of self-capped ZnS QDs isolated at a graded subjected dose of 50 (sample A1), 150 (sample A2) and 200 kGy (sample A3) on a solution of 2-(dimethylamino)ethanethiolate of zinc(II) (**1**) in acetonitrile at a dose rate of 6.6 kGy h^−1^.

The band gap values determined for A1, A2 and A3 are 3.95, 3.89 and 3.84 eV, respectively (figure 6), and point out a decrease in the band gap with a surge in the γ-radiation dose and hence particle size. This trend in band gap energies is consistent with that observed for the band gap estimated from PL emission maximum of samples in chloroform. However, a lower value of band gap relative to band gap values obtained from Tauc’s plot might be owing to the fact that the emission spectra are usually dependent on polarity and relaxation of the solvent (table 2). However, the difference between band gap values determined from Tauc’s plot and UV–vis *λ*_onset_ data is relatively smaller. A notable blue shift of A1, A2 and A3 band gap values relative to the bulk band gap of ZnS (*E*_*g*_ (bulk) = 3.6 eV) in corroboration with the appearance of exciton features in UV–vis substantiates the quantum confinement effect.

### 3.5. Life time measurements of self-capped ZnS quantum dots

**Figure 7.**
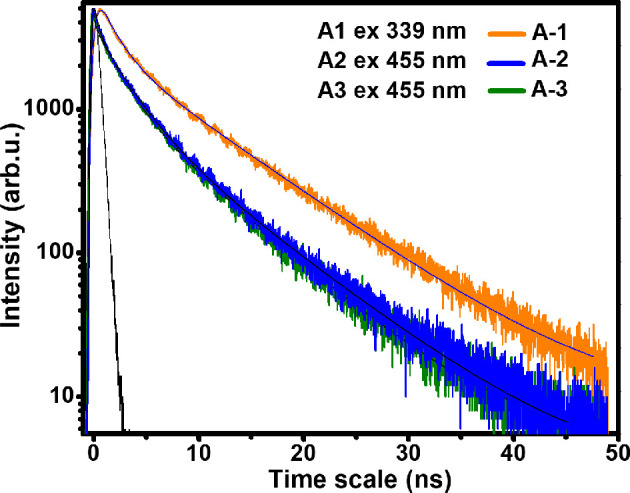
PL decay spectra of A1, A2 and A3 samples of ZnS self-capped QDs.


Time-resolved transient PL decay spectroscopy has been performed on samples A1, A2 and A3 (figure 7) to study PL decay lifetimes corresponding to ZnS QDs by fitting the decay curves using equation (3.5). PL decay profiles of A1 can be fitted with equations (3.5) and (3.6), while A2 and A3 can be fitted with equation (3.6) [37]

(3.5) $$I(t)=A_{1}\cdot exp(-t/\tau_{1})+A_{2}\cdot exp(-t/\tau_{2}),$$

(3.6) $$I(t)=A_{1}\cdot exp(-t/\tau_{1})+A_{2}\cdot exp(-t/\tau_{2})+A_{3}\cdot exp(-t/\tau_{3}),$$

where *τ*_1,_ *τ*_2_ and *τ*_3_ are decay lifetimes of the excited state emission, while *A*_1_, *A*_2_ and *A*_3_ denote the concerning amplitude. The lifetime data of A1, A2 and A3 are listed in table 3. The average lifetime is determined by the following equation (3.7) [38]:

**Table 3.** Lifetime parameters of A1, A2 and A3 samples of ZnS self-capped QDs.

	excitation 339 and 455 nm						
	*τ*_1_ (ns)	*A*_1_ (%)	*τ*_2_ (ns)	*A*_2_ (%)	*τ*_3_ (ns)	*A*_3_ (%)	*τ*_*A*_ (ns)
A1	1.69	22.74	8.36	77.26	—	—	7.98
A2	2.13	35.40	12.54	4.70	6.82	59.90	6.08
A3	2.68	41.09	7.75	51.57	34.88	7.33	6.62


(3.7) $$\tau_{A}=\frac{A_{1}\tau_{1}^{2}+A_{2}\tau_{2}^{2}}{A_{1}\tau_{1}+A_{2}\tau_{2}}.$$

Long lifetime components determined from the PL decay curves range from 6.82 to 34.88 ns, whereas short lifetime components are in the range of 1.69−2.68 ns. The domination of slower PL decay components for all the samples may be owing to the recombination of electrons and holes from shallow trap states [39]. While faster decay components—which are smaller in fraction—might be either, owing to band edge emission or owing to charge carrier recombination at the surface. The tendency in experimental lifetimes is in line with the variation in degree of carrier quenching happening at the surface and the bulk of the QDs. Furthermore, unnoticed band edge contribution in the steady-state spectra [40,41] points out the participation of a highly efficient trapping channel in the trap state emission. Nevertheless, the chance of the band edge emission cannot be excluded completely, as it might have been screened owing to the intense trap emission. Such an observation has been seen in CdS QDs [42].

## 4. Conclusions

A new approach named the γ-assisted molecular template method for the synthesis of hexagonal ZnS QDs by the γ-irradiation of SSMP, 2-(dimethylamino)ethanethiolate of zinc(II) (scheme 1), in CH_3_CN/H_2_O has been presented in the current study. Moreover, the mechanism for the generation of self-capped ZnS QDs has also been explained. Also, size-dependent PL emission and optical band gap tuning of self-capped ZnS QDs were investigated in the current study. The self-capped ZnS QDs of size 3.9−9.5 nm exhibit PL emission tunability in the range of 417–537 nm. These ZnS QDs also demonstrate size-tunable PLQYs to the tune of 10–24%. In brief, γ-irradiation of dimethylamineethane thiolate of metal marks an interesting, facile and prospective approach to synthesizing luminescent metal chalcogenide nanomaterials with tunable optical properties for optoelectronic applications.

## Data Availability

The data supporting this article have been uploaded as part of the online supplementary material [43]. This includes synthesis of precursor and additional figures, etc., which are available.
